# Crystal structure and mol­ecular docking study of (*E*)-2-{[(*E*)-2-hy­droxy-5-methyl­benzyl­idene]hydrazinyl­idene}-1,2-di­phenyl­ethan-1-one

**DOI:** 10.1107/S2056989021005442

**Published:** 2021-05-28

**Authors:** Sevgi Kansiz, Digdem Tatlidil, Necmi Dege, Feyzi Alkim Aktas, Samir Osman Mohammed Al-Asbahy, Aysen Alaman Agar

**Affiliations:** a Samsun University, Faculty of Engineering, Department of Fundamental Sciences, 55420, Samsun, Turkey; b Ondokuz Mayıs University, Faculty of Arts and Sciences, Department of Chemistry, 55139, Samsun, Turkey; c Ondokuz Mayıs University, Faculty of Arts and Sciences, Department of Physics, 55139, Samsun, Turkey; d Samsun University, Faculty of Engineering, Biomedical Engineering, 55420, Samsun, Turkey; e Ibb University, Science College, Department of Physics, Ibb, Yemen; f Aljanad University for Science & Technology, Engineering College, Taiz, Yemen

**Keywords:** crystal structure, Schiff base, di­phenyl­ethan, mol­ecular docking, COVID-19 main protease

## Abstract

The title compound, C_22_H_18_N_2_O_2_, is a Schiff base that exists in the phenol–imine tautomeric form and adopts an *E* configuration with respect to the C=N bond. The mol­ecular structure is stabilized by an O—H⋯N hydrogen bond, forming an *S*(6) ring motif. In the crystal, pairs of C—H⋯O hydrogen bonds link the mol­ecules to form inversion dimers. Weak π–π stacking inter­actions along the *a-*axis direction provide additional stabilization of the crystal structure. The mol­ecule is non-planar, the aromatic ring of the benzaldehyde residue being nearly perpendicular to the phenyl and 4-methyl­phenol rings with dihedral angles of 88.78 (13) and 82.26 (14)°, respectively. A mol­ecular docking study between the title mol­ecule and the COVID-19 main protease (PDB ID: 6LU7) was performed, showing that it is a potential agent because of its affinity and ability to adhere to the active sites of the protein.

## Abstract 2

The title compound, C_22_H_18_N_2_O_2_, is a Schiff base that exists in the phenol–imine tautomeric form and adopts an *E* configuration. The mol­ecular structure is stabilized by an O—H⋯N hydrogen bond, forming an *S*(6) ring motif.

## Chemical context   

Schiff bases have wide applications inter­ests as corrosion inhibitors (Antonijevic & Petrovic, 2008 ▸), biologically active materials (Al Zoubi, 2013 ▸) and thermostable systems (Destri *et al.*, 1998 ▸). The optical and semiconducting phenomena of the azomethine linkage group have been also widely investigated as a result of their photo-efficiency, with wavelengths depending on the chemical architecture of the Schiff-base mol­ecules (Iwan & Sek, 2008 ▸). Schiff bases have significant importance in the development of metal complexes, because Schiff base ligands are potentially capable of forming stable complexes by coordination of metal ions *via* their oxygen and nitro­gen donors (Ebrahimipour *et al.*, 2012 ▸). Hydrazine, hydrazone and hydrazide derivatives are relatively scarce in nature and have been isolated from plants, marine organisms and microorganisms. These compounds exhibit remarkable structural diversity and relevant biological activities (Le Goff & Ouazzani, 2014 ▸). Salicyl­aldehyde complexes with transition metals have worked as anti­malarial and anti­leukemic agents (Scovill *et al.*, 1982 ▸). In this study, a new Schiff base with potential biological character, (*E*)-2-{[(*E*)-2-hy­droxy-5-meth­yl­benzyl­idene]hydrazineyl­idene}-1,2-di­phenyl­ethan-1-one, was obtained in crystalline form from the reaction of 2-hy­droxy-5-methyl­benzaldehyde with (*E*)-2-hydrazineyl­idene-1,2-di­phenyl­ethan-1-one. We report here the synthesis, crystal and mol­ecular structure of the title compound. We have also performed a mol­ecular docking study to determine possible inter­molecular inter­actions between the COVID-19 main protease (PDB ID: 6LU7) and the title compound.
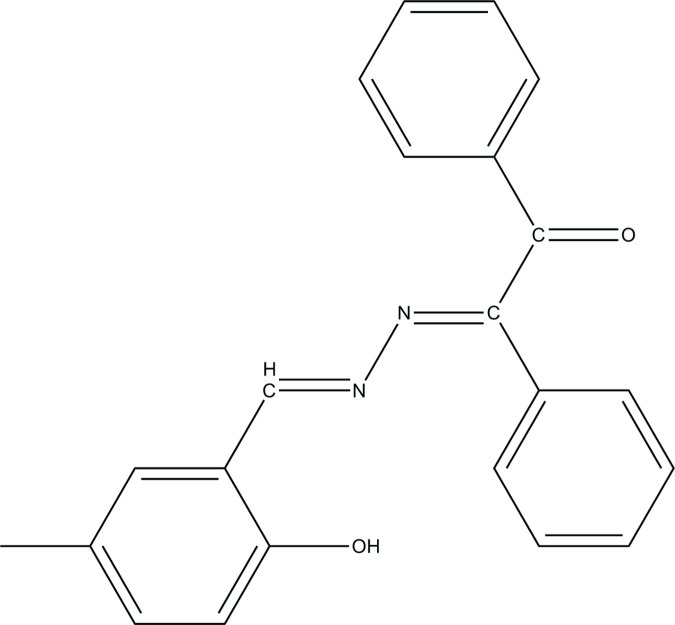


## Structural commentary   

**Figure 1:**
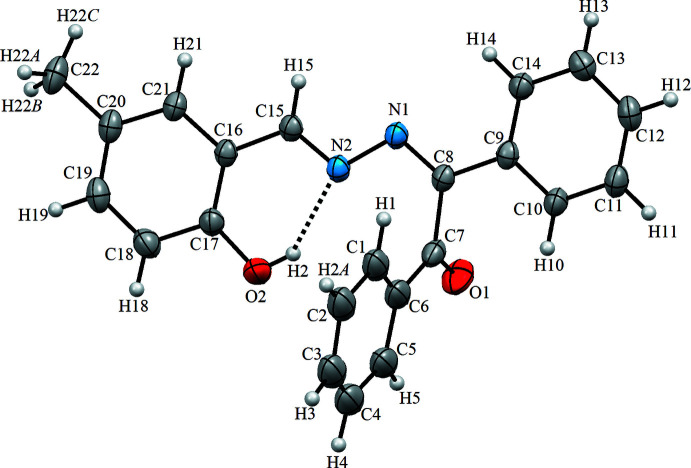
The mol­ecular structure of the title compound with the atom-labelling scheme. Displacement ellipsoids are drawn at the 40% probability level. Dashed lines denote the intra­molecular O—H⋯N hydrogen bonds forming an *S*(6) ring motif.

The asymmetric unit of the title structure contains one mol­ecule (Fig. 1 ▸), which crystallizes in the phenol–imine tautomeric form with an *E* configuration for the imine functionality. The hy­droxy H atom is involved in a strong intra­molecular O—H⋯N hydrogen bond, forming an *S*(6) ring motif, which stabilizes the mol­ecular structure. The di­benzyl­idene hydrazine unit is approximately planar with the dihedral angle formed by the two terminal phenyl rings of 7.62 (15)°. On the other hand, the mol­ecule is non-planar, because the C1–C6 ring is nearly perpendicular to the C9–C14 and C16–C21 rings with dihedral angles of 88.78 (13) and 82.26 (14)°, respectively. The C17—O2, C15—N2 and C15—C16 bond lengths in the mol­ecule are 1.359 (5), 1.287 (5), and 1.452 (5) Å, respectively. These results suggest single-bond character for C17—O2 and C15—C16 and double-bond character for the C15—N2 bond as expected for a phenol–imine structure (Kaştaş *et al.*, 2020 ▸). The bond lengths and angles in the title mol­ecule agree reasonably well with those found in closely related structures (Bouchama *et al.*, 2015 ▸; Wieland *et al.*, 2011 ▸). Based on the refinement parameters, the tautomeric form of the compound is the phenol–imine form in which the tautomeric proton (H2) is located on the phenolic oxygen atom (O2). The distance of 2.650 (5) Å between the nitro­gen and the oxygen atoms show that the mol­ecule has a strong O—H⋯N intra­molecular hydrogen bond, forming an *S*(6) ring motif.

## Supra­molecular features   

**Figure 2:**
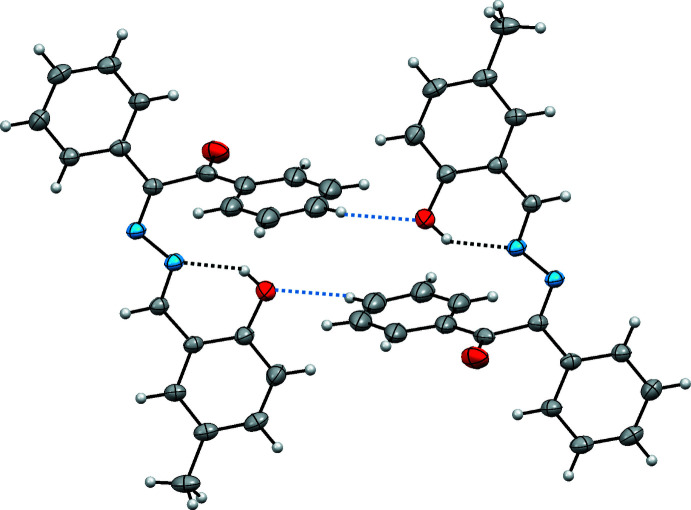
A view of the crystal packing of the title compound. Blue dashed lines denote the inter­molecular C3—H3⋯O2 hydrogen bonds forming an inversion dimer (Table 1 ▸).

In the crystal, mol­ecules are linked by pairs of C3—H3⋯O2 hydrogen bonds, forming inversion dimers with an 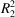
(11) ring motif (Table 1 ▸ and Fig. 2 ▸). There are also weak π–π inter­actions [*Cg*2⋯*Cg*3(−*x*, −*y*, −*z*) = 3.909 (2) Å; *Cg*2 and *Cg*3 are the centroids of the C9–C14 and C16–C21 rings, respectively] that stabilize the crystal structure, forming a three-dimensional network.

## Database survey   

A search of the Cambridge Structural Database (CSD, version 5.42, update November 2020; Groom *et al.*, 2016 ▸) for the (benzyl­idenehydrazono)-1,2-di­phenyl­ethanone skeleton revealed 14 similar compounds. In MOZZEH01 (Marcel *et al.*, 2011 ▸), the C=N [1.291 (2) Å], N—N [1.414 (2) Å] and C=O bond lengths [1.235 (1) Å] are within the ranges observed for the title compound. The C7—C8 bond distance of 1.523 (2) Å corresponding to the value expected for a C*sp* ^2^—C*sp* ^2^ single bond, is slightly longer than observed for the title compound [1.472 (5) Å]. This bond length is shorter than in NOTZIH [1.528 (3) Å; Bouchama *et al.*, 2015 ▸]. In HULFAX (Elmacı *et al.*, 2015 ▸), the C15—N2 bond length [1.276 (4) Å] is typical of for an azomethine C=N bond and shorter than in the title compound [1.287 (5) Å]. In MOZZEH (Patra *et al.*, 2009 ▸) and MUBTUZ (Patra & Ng, 2009 ▸), the di­methyl­ene hydrazine (—C=N—N=C—) units are approximately planar, the torsion angles around the N—N bond being 177.82 (12) and 162.2 (6)°, respectively. Although these values are comparable to the title compound, they are slightly smaller than 178.3 (3)°. In LOTKEN (Yahyaoui *et al.*, 2019 ▸), N—N (hydrazone) distances are within the range of typical single bond [1.398 (6)–1.4077 (16) Å and the C=N bonds in the hydrazone units are between 1.2893 (19) and 1.3014 (18) Å]. The torsion angles involving the —N=C— vary between −171.02 and −179.90°. All these values are similar to those observed in the title compound.

## Mol­ecular docking study   

**Figure 3:**
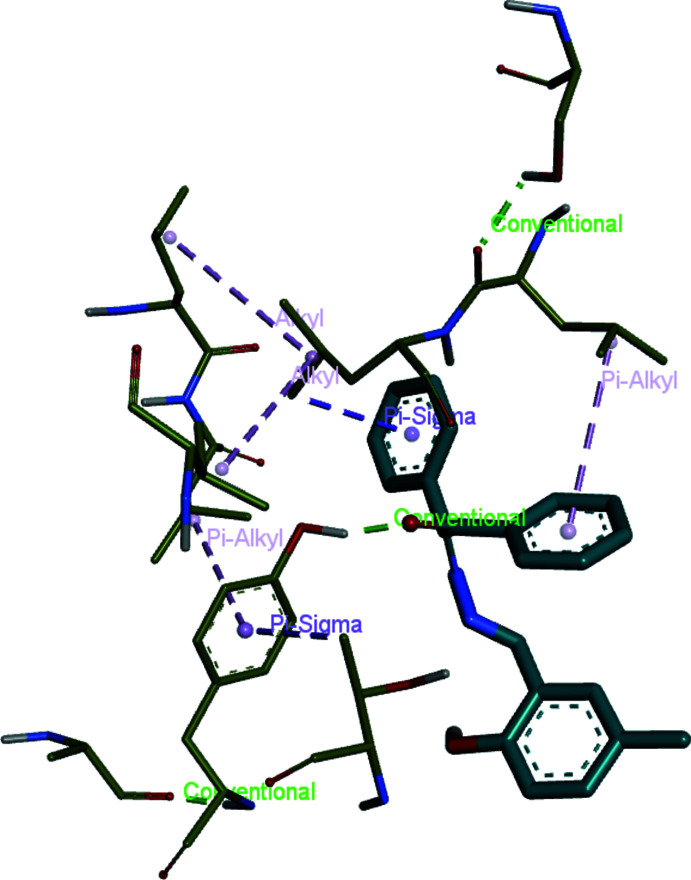
Three-dimensional visualization of the inter­molecular inter­actions for the best binding pose of the title compound docking with 6LU7.

**Figure 4:**
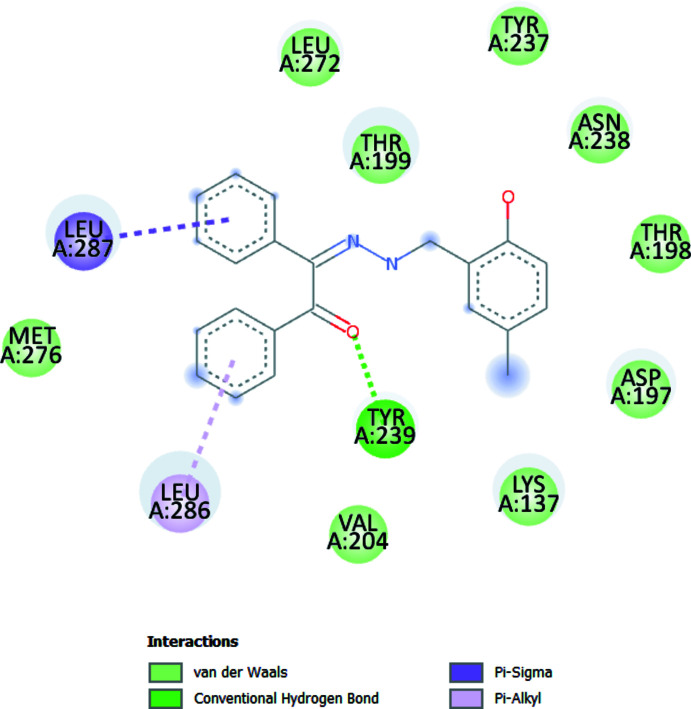
Two-dimensional visuals of the inter­molecular inter­actions for the best binding pose of the title compound docking with 6LU7.

**Figure 5:**
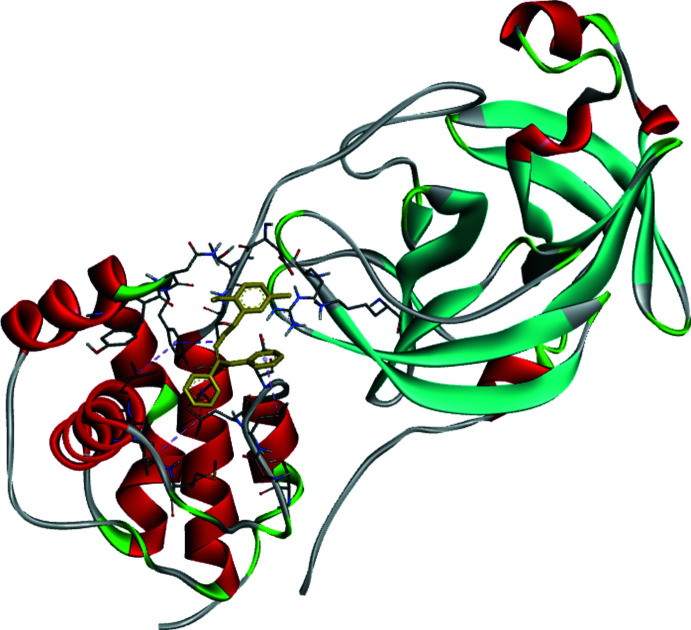
Three-dimensional conformation of the complex of the title compound with 6LU7.

Mol­ecular docking is a crucial method for investigating the inter­action between small mol­ecules and macromolecules. Inter­molecular contacts that occur between a ligand and a protein are evaluated by mol­ecular docking. In summary, this method is one of the major approaches to estimate the binding area where the ligand connects with the protein. In this study, *AutoDockVina* (Trott & Olson, 2010 ▸) was utilized for predicting binding sites between the title mol­ecule and 6LU7. 6LU7 is a main protease of COVID-19, and can be efficient for drug design to treat ailments (Jin *et al.*, 2020 ▸). The three-dimensional structure of 6LU7 was received from the Protein Data Bank (PDB). Before the computation, the protein must be prepared for efficient insertion. Therefore, all water mol­ecules and ligands were removed from protein active sites. LYS102, VAL104, GLN110, THR111, ASN151, ASP153 and SER158 were defined as active areas. According to these active sites, grid box dimensions were determined to be 100 × 100 × 95 Å. In addition, ‘*x*, *y*, *z*’ centers were adjusted to be −20.378, 27.848, 69.124, respectively, and then the 6LU7 protein was saved in PDBQT format for calculations. In the next step of the experiment, rotatable angles for coupling structures were identified and recorded in PDBQT format. *Discovery Studio Visualizer* (Biovia, 2017 ▸) was used for observations and preparations. All docking calculations were computed with *AutoDockVina*. Twenty variable adherences were decided by *AutoDockVina* for the ligands connected to the receptor of the protein. The best affinity energy was observed in the first calculation, of −7.2 kcal mol^−1^. The bonding type of inter­action is demonstrated in Fig. 3 ▸. The 2D and 3D visuals of the inter­molecular inter­actions for the best binding pose of the title compound docked into macromolecule 6LU7 can be seen in Fig. 4 ▸. In addition, the docking conformation is shown in Fig. 5 ▸. As a consequence, the title compound could be a potential mol­ecule for drug design to treat severe acute respiratory syndrome resulting from the novel corona virus SARS CoV2 because of its affinity and ability suitable to adhere to active sites of the protein.

## Synthesis and crystallization   

(*E*)-2-{[(*E*)-2-Hy­droxy-5-methyl­benzyl­idene]hydrazineyl­id­ene}-1,2-di­phenyl­ethan-1-one was prepared by refluxing a mixture of a solution containing 2-hy­droxy-5-methyl­benzaldehyde (0.02 mmol) in ethanol (20 mL) and a solution containing (*E*)-2-hydrazineyl­idene-1,2-di­phenyl­ethan-1-one (0.02 mmol) in ethanol (20 mL). The reaction mixture was stirred for 5 h under reflux. The obtained crystalline material was washed with ethanol and dried at room temperature. Single crystals of the title compound for X-ray analysis were obtained by slow evaporation of an ethanol solution.

## Refinement   

Crystal data, data collection and structure refinement details are summarized in Table 2 ▸. The O-bound H atom was located in a difference-Fourier map and refined with O—H = 0.82 Å and *U*_iso_(H) = 1.5*U*_eq_(O). The C-bound H atoms were positioned geometrically and refined using a riding model with C—H = 0.93 and *U*_iso_(H) = 1.2*U*_eq_(C) for aromatic H atoms, and with C—H = 0.96 Å and *U*_iso_(H) = 1.5*U*_eq_(C) for methyl H atoms.

## Supplementary Material

Crystal structure: contains datablock(s) I. DOI: 10.1107/S2056989021005442/yk2152sup1.cif

Structure factors: contains datablock(s) I. DOI: 10.1107/S2056989021005442/yk2152Isup2.hkl

Supporting information file. DOI: 10.1107/S2056989021005442/yk2152Isup3.cml

CCDC reference: 2085577

Additional supporting information:  crystallographic information; 3D view; checkCIF report

## Figures and Tables

**Table 1:** Hydrogen-bond geometry (Å, °)

*D*—H⋯*A*	*D*—H	H⋯*A*	*D*⋯*A*	*D*—H⋯*A*
O2—H2⋯N2	0.82	1.94	2.650 (5)	145
C3—H3⋯O2^i^	0.93	2.54	3.434 (8)	162

Symmetry code: (i) -x+1, -y+2, -z+1.

**Table 2:** Experimental details

Crystal data	
Chemical formula	C_22_H_18_N_2_O_2_
*M*_r_	342.38
Crystal system, space group	Monoclinic, *P*2_1_/*c*
Temperature (K)	296
*a*, *b*, *c* (Å)	7.4089 (6), 11.4544 (14), 21.9491 (17)
β (°)	97.814 (6)
*V* (Å^3^)	1845.4 (3)
*Z*	4
Radiation type	Mo *K*α
μ (mm^−1^)	0.08
Crystal size (mm)	0.71 × 0.49 × 0.21

Data collection	
Diffractometer	Stoe *IPDS* 2
Absorption correction	Integration (*X-RED32*; Stoe & Cie, 2002 ▸)
*T*_min_, *T*_max_	0.952, 0.987
No. of measured, independent and observed [*I* > 2σ(*I*)] reflections	8376, 3262, 1801
*R*_int_	0.037
(sin θ/λ)_max_ (Å^−1^)	0.596

Refinement	
*R*[*F* ^2^ > 2σ(*F* ^2^)], *wR*(*F* ^2^), *S*	0.085, 0.287, 1.11
No. of reflections	3262
No. of parameters	237
No. of restraints	84
H-atom treatment	H-atom parameters constrained
Δρ_max_, Δρ_min_ (e Å^−3^)	0.50, −0.45

Computer programs: *X-AREA* and *X-RED* (Stoe & Cie, 2002 ▸), *SHELXT2018/3* (Sheldrick, 2015*a* ▸), *SHELXL2018/3* (Sheldrick, 2015*b* ▸), *PLATON* (Spek, 2020 ▸) and *WinGX* (Farrugia, 2012 ▸).

## supplementary crystallographic information

### Crystal data

**Table:**

C_22_H_18_N_2_O_2_	*F*(000) = 720
*M**_r_* = 342.38	*D*_x_ = 1.232 Mg m^−^^3^
Monoclinic, *P*2_1_/*c*	Mo *K*α radiation, λ = 0.71073 Å
*a* = 7.4089 (6) Å	Cell parameters from 7807 reflections
*b* = 11.4544 (14) Å	θ = 1.8–29.1°
*c* = 21.9491 (17) Å	µ = 0.08 mm^−^^1^
β = 97.814 (6)°	*T* = 296 K
*V* = 1845.4 (3) Å^3^	Prism, orange
*Z* = 4	0.71 × 0.49 × 0.21 mm

### Data collection

**Table:**

Stoe IPDS 2 diffractometer	3262 independent reflections
Radiation source: sealed X-ray tube, 12 x 0.4 mm long-fine focus	1801 reflections with *I* > 2σ(*I*)
Plane graphite monochromator	*R*_int_ = 0.037
Detector resolution: 6.67 pixels mm^-1^	θ_max_ = 25.1°, θ_min_ = 1.9°
rotation method scans	*h* = −7→8
Absorption correction: integration (X-RED32; Stoe & Cie, 2002)	*k* = −13→12
*T*_min_ = 0.952, *T*_max_ = 0.987	*l* = −26→26
8376 measured reflections	

### Refinement

**Table:**

Refinement on *F*^2^	84 restraints
Least-squares matrix: full	Hydrogen site location: inferred from neighbouring sites
*R*[*F*^2^ > 2σ(*F*^2^)] = 0.085	H-atom parameters constrained
*wR*(*F*^2^) = 0.287	*w* = 1/[σ^2^(*F*_o_^2^) + (0.1388*P*)^2^ + 0.7696*P*] where *P* = (*F*_o_^2^ + 2*F*_c_^2^)/3
*S* = 1.11	(Δ/σ)_max_ < 0.001
3262 reflections	Δρ_max_ = 0.50 e Å^−^^3^
237 parameters	Δρ_min_ = −0.45 e Å^−^^3^

### Special details

**Table:**

Geometry. All esds (except the esd in the dihedral angle between two l.s. planes) are estimated using the full covariance matrix. The cell esds are taken into account individually in the estimation of esds in distances, angles and torsion angles; correlations between esds in cell parameters are only used when they are defined by crystal symmetry. An approximate (isotropic) treatment of cell esds is used for estimating esds involving l.s. planes.

### Fractional atomic coordinates and isotropic or equivalent isotropic displacement parameters (Å^2^)

**Table:**

	*x*	*y*	*z*	*U*_iso_*/*U*_eq_	
N2	0.8073 (5)	0.5988 (3)	0.50905 (13)	0.0632 (9)	
N1	0.7264 (5)	0.5098 (3)	0.47015 (13)	0.0650 (9)	
O2	0.9055 (6)	0.8135 (3)	0.54443 (14)	0.0968 (12)	
H2	0.866243	0.762928	0.519659	0.145*	
O1	0.8305 (6)	0.6961 (4)	0.37321 (16)	0.1135 (14)	
C16	0.9521 (5)	0.6438 (4)	0.61058 (16)	0.0587 (10)	
C9	0.5718 (5)	0.4721 (4)	0.36834 (16)	0.0604 (10)	
C15	0.8727 (5)	0.5634 (4)	0.56320 (16)	0.0605 (10)	
H15	0.869084	0.484218	0.572232	0.073*	
C8	0.6674 (5)	0.5488 (3)	0.41603 (16)	0.0587 (10)	
C21	1.0127 (6)	0.6008 (4)	0.66984 (15)	0.0647 (11)	
H21	1.008655	0.520813	0.676726	0.078*	
C14	0.5475 (6)	0.3537 (4)	0.37911 (18)	0.0701 (12)	
H14	0.592287	0.321938	0.417175	0.084*	
C20	1.0782 (6)	0.6731 (5)	0.71822 (17)	0.0722 (12)	
C7	0.7011 (7)	0.6748 (4)	0.39951 (17)	0.0729 (12)	
C17	0.9626 (6)	0.7633 (4)	0.59982 (19)	0.0704 (12)	
C10	0.5008 (6)	0.5172 (4)	0.31111 (17)	0.0740 (12)	
H10	0.513452	0.596328	0.303221	0.089*	
C13	0.4574 (7)	0.2826 (4)	0.3337 (2)	0.0806 (13)	
H13	0.442179	0.203596	0.341196	0.097*	
C12	0.3900 (7)	0.3298 (5)	0.2771 (2)	0.0809 (14)	
H12	0.329630	0.282317	0.246532	0.097*	
C19	1.0877 (7)	0.7906 (5)	0.7057 (2)	0.0844 (15)	
H19	1.134050	0.840615	0.737345	0.101*	
C11	0.4120 (7)	0.4460 (5)	0.26600 (19)	0.0832 (14)	
H11	0.366915	0.477135	0.227836	0.100*	
C18	1.0317 (7)	0.8376 (4)	0.6481 (2)	0.0874 (14)	
H18	1.039779	0.917581	0.641626	0.105*	
C22	1.1374 (8)	0.6258 (6)	0.78220 (19)	0.0994 (18)	
H22A	1.044825	0.641737	0.807719	0.149*	
H22B	1.249168	0.662613	0.799408	0.149*	
H22C	1.155645	0.543006	0.780046	0.149*	
C3	0.3300 (11)	0.9322 (6)	0.4407 (3)	0.1213 (13)	
H3	0.245666	0.989489	0.447121	0.146*	
C2	0.2919 (10)	0.8188 (5)	0.4544 (3)	0.1180 (12)	
H2A	0.188797	0.799054	0.472040	0.142*	
C6	0.5724 (10)	0.7645 (5)	0.4144 (2)	0.1031 (12)	
C1	0.4177 (10)	0.7334 (5)	0.4404 (2)	0.1081 (12)	
H1	0.396996	0.655268	0.448600	0.130*	
C5	0.6069 (10)	0.8824 (5)	0.4039 (2)	0.1137 (12)	
H5	0.710653	0.905524	0.387565	0.136*	
C4	0.4790 (11)	0.9644 (6)	0.4189 (2)	0.1204 (13)	
H4	0.500278	1.043459	0.413196	0.144*	

### Atomic displacement parameters (Å^2^)

**Table:**

	*U*^11^	*U*^22^	*U*^33^	*U*^12^	*U*^13^	*U*^23^
N2	0.066 (2)	0.068 (2)	0.0531 (17)	0.0089 (17)	−0.0017 (14)	−0.0065 (15)
N1	0.073 (2)	0.069 (2)	0.0499 (17)	0.0104 (17)	−0.0016 (15)	−0.0052 (15)
O2	0.137 (3)	0.0681 (19)	0.076 (2)	0.010 (2)	−0.0158 (19)	0.0019 (16)
O1	0.123 (3)	0.137 (3)	0.086 (2)	−0.046 (3)	0.033 (2)	0.010 (2)
C16	0.054 (2)	0.070 (3)	0.0515 (19)	0.0107 (19)	0.0062 (16)	−0.0051 (17)
C9	0.054 (2)	0.076 (3)	0.0511 (19)	0.007 (2)	0.0080 (16)	−0.0057 (18)
C15	0.059 (2)	0.067 (2)	0.055 (2)	0.0119 (19)	0.0068 (17)	−0.0041 (17)
C8	0.055 (2)	0.071 (3)	0.0500 (19)	0.0077 (19)	0.0063 (16)	0.0002 (17)
C21	0.061 (3)	0.083 (3)	0.050 (2)	0.001 (2)	0.0076 (17)	−0.0030 (18)
C14	0.077 (3)	0.073 (3)	0.058 (2)	0.006 (2)	0.004 (2)	−0.002 (2)
C20	0.058 (3)	0.103 (4)	0.054 (2)	0.003 (2)	0.0051 (18)	−0.011 (2)
C7	0.084 (3)	0.085 (3)	0.047 (2)	−0.020 (3)	0.001 (2)	0.0051 (19)
C17	0.078 (3)	0.070 (3)	0.061 (2)	0.012 (2)	0.000 (2)	−0.007 (2)
C10	0.087 (3)	0.082 (3)	0.051 (2)	0.003 (2)	0.0026 (19)	−0.003 (2)
C13	0.083 (3)	0.076 (3)	0.082 (3)	0.002 (2)	0.010 (2)	−0.016 (2)
C12	0.073 (3)	0.101 (4)	0.068 (3)	0.006 (3)	0.003 (2)	−0.029 (3)
C19	0.077 (3)	0.105 (4)	0.068 (3)	−0.005 (3)	−0.001 (2)	−0.026 (3)
C11	0.087 (4)	0.105 (4)	0.054 (2)	0.008 (3)	−0.002 (2)	−0.009 (2)
C18	0.098 (4)	0.074 (3)	0.087 (3)	0.001 (3)	0.001 (3)	−0.016 (2)
C22	0.097 (4)	0.147 (5)	0.052 (2)	−0.010 (3)	0.000 (2)	−0.007 (3)
C3	0.163 (3)	0.104 (2)	0.0846 (19)	0.038 (2)	−0.0283 (19)	−0.0113 (17)
C2	0.157 (3)	0.105 (2)	0.0817 (18)	0.036 (2)	−0.0232 (18)	−0.0129 (17)
C6	0.153 (3)	0.0836 (19)	0.0617 (17)	0.024 (2)	−0.0240 (17)	−0.0021 (16)
C1	0.150 (3)	0.0933 (19)	0.0707 (18)	0.033 (2)	−0.0220 (18)	−0.0100 (16)
C5	0.165 (3)	0.0914 (19)	0.0731 (18)	0.022 (2)	−0.0253 (18)	0.0004 (16)
C4	0.169 (3)	0.097 (2)	0.0819 (19)	0.028 (2)	−0.0292 (19)	−0.0017 (17)

### Geometric parameters (Å, º)

**Table:**

N2—C15	1.287 (5)	C10—H10	0.9300
N2—N1	1.411 (4)	C13—C12	1.384 (6)
N1—C8	1.289 (4)	C13—H13	0.9300
O2—C17	1.359 (5)	C12—C11	1.366 (7)
O2—H2	0.8200	C12—H12	0.9300
O1—C7	1.209 (5)	C19—C18	1.384 (7)
C16—C17	1.393 (6)	C19—H19	0.9300
C16—C21	1.406 (5)	C11—H11	0.9300
C16—C15	1.452 (5)	C18—H18	0.9300
C9—C14	1.393 (6)	C22—H22A	0.9600
C9—C10	1.393 (5)	C22—H22B	0.9600
C9—C8	1.472 (5)	C22—H22C	0.9600
C15—H15	0.9300	C3—C4	1.314 (9)
C8—C7	1.518 (6)	C3—C2	1.372 (9)
C21—C20	1.382 (6)	C3—H3	0.9300
C21—H21	0.9300	C2—C1	1.413 (8)
C14—C13	1.386 (6)	C2—H2A	0.9300
C14—H14	0.9300	C6—C1	1.393 (9)
C20—C19	1.377 (7)	C6—C5	1.399 (8)
C20—C22	1.514 (6)	C1—H1	0.9300
C7—C6	1.469 (7)	C5—C4	1.405 (9)
C17—C18	1.402 (6)	C5—H5	0.9300
C10—C11	1.379 (6)	C4—H4	0.9300
			
C15—N2—N1	114.0 (3)	C11—C12—C13	120.2 (4)
C8—N1—N2	111.7 (3)	C11—C12—H12	119.9
C17—O2—H2	109.5	C13—C12—H12	119.9
C17—C16—C21	118.9 (4)	C20—C19—C18	123.1 (4)
C17—C16—C15	121.9 (3)	C20—C19—H19	118.5
C21—C16—C15	119.2 (4)	C18—C19—H19	118.5
C14—C9—C10	118.2 (4)	C12—C11—C10	120.3 (4)
C14—C9—C8	121.4 (3)	C12—C11—H11	119.8
C10—C9—C8	120.4 (4)	C10—C11—H11	119.8
N2—C15—C16	121.8 (4)	C19—C18—C17	119.1 (5)
N2—C15—H15	119.1	C19—C18—H18	120.4
C16—C15—H15	119.1	C17—C18—H18	120.4
N1—C8—C9	121.2 (4)	C20—C22—H22A	109.5
N1—C8—C7	120.3 (3)	C20—C22—H22B	109.5
C9—C8—C7	118.5 (3)	H22A—C22—H22B	109.5
C20—C21—C16	122.4 (4)	C20—C22—H22C	109.5
C20—C21—H21	118.8	H22A—C22—H22C	109.5
C16—C21—H21	118.8	H22B—C22—H22C	109.5
C13—C14—C9	120.7 (4)	C4—C3—C2	123.6 (7)
C13—C14—H14	119.7	C4—C3—H3	118.2
C9—C14—H14	119.7	C2—C3—H3	118.2
C19—C20—C21	117.0 (4)	C3—C2—C1	116.5 (7)
C19—C20—C22	121.3 (4)	C3—C2—H2A	121.8
C21—C20—C22	121.7 (5)	C1—C2—H2A	121.8
O1—C7—C6	123.2 (5)	C1—C6—C5	119.6 (6)
O1—C7—C8	118.2 (5)	C1—C6—C7	120.5 (5)
C6—C7—C8	118.6 (4)	C5—C6—C7	119.9 (7)
O2—C17—C16	123.4 (4)	C6—C1—C2	121.0 (6)
O2—C17—C18	117.2 (4)	C6—C1—H1	119.5
C16—C17—C18	119.5 (4)	C2—C1—H1	119.5
C11—C10—C9	120.9 (5)	C6—C5—C4	117.6 (7)
C11—C10—H10	119.6	C6—C5—H5	121.2
C9—C10—H10	119.6	C4—C5—H5	121.2
C12—C13—C14	119.7 (5)	C3—C4—C5	121.6 (7)
C12—C13—H13	120.1	C3—C4—H4	119.2
C14—C13—H13	120.1	C5—C4—H4	119.2
			
C15—N2—N1—C8	−178.3 (3)	C14—C9—C10—C11	−1.2 (6)
N1—N2—C15—C16	−176.5 (3)	C8—C9—C10—C11	179.6 (4)
C17—C16—C15—N2	−0.4 (6)	C9—C14—C13—C12	−0.3 (7)
C21—C16—C15—N2	176.7 (4)	C14—C13—C12—C11	−0.1 (7)
N2—N1—C8—C9	−177.8 (3)	C21—C20—C19—C18	1.5 (7)
N2—N1—C8—C7	4.3 (5)	C22—C20—C19—C18	−178.7 (5)
C14—C9—C8—N1	−2.4 (6)	C13—C12—C11—C10	−0.2 (7)
C10—C9—C8—N1	176.7 (4)	C9—C10—C11—C12	0.9 (7)
C14—C9—C8—C7	175.5 (4)	C20—C19—C18—C17	−0.4 (8)
C10—C9—C8—C7	−5.3 (6)	O2—C17—C18—C19	178.6 (4)
C17—C16—C21—C20	1.7 (6)	C16—C17—C18—C19	−0.1 (7)
C15—C16—C21—C20	−175.5 (4)	C4—C3—C2—C1	−3.8 (8)
C10—C9—C14—C13	0.9 (6)	O1—C7—C6—C1	174.8 (4)
C8—C9—C14—C13	−179.9 (4)	C8—C7—C6—C1	−3.2 (6)
C16—C21—C20—C19	−2.1 (6)	O1—C7—C6—C5	−6.6 (7)
C16—C21—C20—C22	178.0 (4)	C8—C7—C6—C5	175.3 (4)
N1—C8—C7—O1	96.0 (5)	C5—C6—C1—C2	2.1 (7)
C9—C8—C7—O1	−81.9 (5)	C7—C6—C1—C2	−179.4 (4)
N1—C8—C7—C6	−85.9 (5)	C3—C2—C1—C6	0.5 (7)
C9—C8—C7—C6	96.2 (4)	C1—C6—C5—C4	−1.6 (7)
C21—C16—C17—O2	−179.2 (4)	C7—C6—C5—C4	179.9 (4)
C15—C16—C17—O2	−2.1 (7)	C2—C3—C4—C5	4.4 (9)
C21—C16—C17—C18	−0.5 (6)	C6—C5—C4—C3	−1.5 (8)
C15—C16—C17—C18	176.6 (4)		

### Hydrogen-bond geometry (Å, º)

**Table:**

*D*—H···*A*	*D*—H	H···*A*	*D*···*A*	*D*—H···*A*
O2—H2···N2	0.82	1.94	2.650 (5)	145
C3—H3···O2^i^	0.93	2.54	3.434 (8)	162

Symmetry code: (i) −*x*+1, −*y*+2, −*z*+1.
